# A phase 1 study to assess the safety, tolerability and effectiveness of PV-10 (Rose Bengal Sodium) in neuroendocrine tumours metastatic to the liver

**DOI:** 10.1038/s41416-025-02976-9

**Published:** 2025-03-26

**Authors:** Timothy Price, Laura Depauw, Gabrielle Cehic, Eric Wachter, Ruben Sebben, Jessica Reid, Susan Neuhaus, Anas Alawawdeh, Ian D. Kirkwood, Rahul Solanki, Mark McGregor, Lisa Leopardi, Dominic Rodrigues, Guy Maddern

**Affiliations:** 1 The Queen Elizabeth Hospital, Woodville, SA Australia; 2 University of Adelaide, Adelaide, SA Australia; 3 Provectus Biopharmaceuticals, Inc, Knoxville, TN USA; 4 Royal Adelaide Hospital, Adelaide, SA Australia

## Abstract

**Background:**

Metastatic neuroendocrine neoplasms (mNEN) require new treatment options. Intralesional (IL) PV-10 is an autolytic chemotherapy that may elicit an adaptive immune response.

**Methods:**

This phase 1 study evaluated IL PV-10 administered percutaneously to hepatic lesions in patients with progressive mNEN. IL PV-10 was injected in a single lesion per treatment cycle. A treatment cycle could be repeated after ≥ 6 weeks if multiple targetable lesions were present. The primary endpoint was safety.

**Results:**

Twelve patients were enrolled with a median age of 66 years (range 47–79). All patients had progressive disease at enrolment and received prior somatostatin analogues; 10 patients had peptide receptor radionuclide therapy (PRRT) treatment. One lesion was injected per cycle for all 12 patients. Reported grade 3 side effects were photosensitivity (1 patient), face oedema (1 patient), elevated transaminases (1 patient), hypertension (2 patients). Response rate was 42% of injected lesions with patient-level disease control of 84%, PFS 9.4 months and median OS 24.0 months.

**Conclusions:**

IL PV-10 elicited no safety concerns. Encouraging evidence of local and systemic disease control was seen in a heavily pre-treated, progressing mNEN population.

**Clinical Trial Registration number:**

NCT02693067.

## Introduction

### Background

Neuroendocrine neoplasms (NEN) arise from enterochromaffin-like cells that can release hormones into the blood resulting in various symptoms. These cells are able to take up amine precursors, decarboxylate them and secrete the resulting peptide hormones [1]. NEN can be divided into well-differentiated neuroendocrine tumours (NET) and poorly differentiated neuroendocrine carcinomas (NEC). NEN are rare tumours, with NET having an incidence of around 5–7 per 100,000 of the population, whereas NEC only present in 1.5–4.6 per 1,000,000, according to the SEER database.

Whilst many NEN are confined to areas of the gastrointestinal tract, they may also be found in the lungs and rarely in the skin, ovaries or thymus [2]. The location of the tumours is important because some clinical features are unique to the site of origin and both survival and response to treatment are influenced by the primary tumour site [3].

Although metastatic neuroendocrine neoplasms (mNEN) originating in the gastrointestinal tract are frequently slow growing, disease control remains important over time. Treatment options include surgical resection [4], chemoablation [5] or chemoembolization of the hepatic artery [6], systemic therapy with systemic somatostatin analogues (e.g., octreotide [7], lanreotide [8]), selective internal radionuclide therapy (SIRT) [9], chemotherapy or tyrosine kinase inhibitors; and radio-labelled somatostatin analogues (e.g.[^177^Lu]Lu-DOTA-octreotate) also known as peptide receptor radionuclide therapy (PRRT) [4, 10, 11].

Furthermore, symptom control is crucial for functional tumours, as they can cause flushing, diarrhoea, wheezing, palpitations, abdominal cramps, and peripheral oedema, which can often be debilitating when not controlled properly [12]. Mesenteric fibrosis and valvular heart disease can develop as a long-term consequence of uncontrolled functional disease. Treatment aimed at controlling and reducing serotonin (5-hydroxytriptamine) secretion, which has been identified as one of the possible hormones involved in functional syndrome together with histamine, bradykinins, and tachykinins, is important to reduce these late effects [13–17].

## Systemic therapy

Standard treatment options in progressive, metastatic NEN include somatostatin analogues, everolimus, sunitinib and chemotherapy. A meta-analysis concluded that everolimus demonstrated a statistically significant difference in progression free survival (PFS) in all tumour subtypes compared to placebo, however, there was no proven difference in overall survival (OS) [18]. In a phase 2 trial with 50 mNEN patients of gastroenteropancreatic or lung origin, the combination of everolimus 10 mg daily and octreotide LAR 30 mg every 28 days generated an objective response rate (ORR) of 18% and stable disease in 74% of patients [19]. A meta-analysis investigating everolimus in advance pancreatic NEN, found a similar ORR and disease control rate (DCR) of 12% and 73% respectively, and median PFS of 14.7 months [20]. There was no data on OS. Everolimus is known to have considerable side effects, such as mucositis, rash, diarrhoea, thrombocytopenia, anaemia, hyperglycaemia, interstitial lung disease, and even a few cases of lethal pneumonitis have been described [21]. A retrospective analysis of 169 patients with heavily pretreated NEN reported high toxicity rates of everolimus: 85% of patients experienced any adverse event (AE), of which 46.1% were grade 3–4 [22].

Sunitinib has significant activity, particularly in pancreatic NEN. A randomized, double-blind phase 3 trial of sunitinib versus placebo in pancreatic NEN (pNEN) reported that the PFS was significantly longer in the sunitinib group compared to placebo (11.4 months vs 5.5 months; HR 0.42; *P* < 0.001) [23]. The ORR was 9.3% in the sunitinib group versus 0% in placebo, however the trial was discontinued early due to the favourable PFS in the sunitinib group. Because of the high number of censored events, the median OS could not be estimated. The most frequent reported adverse event in the sunitinib group being diarrhoea (59%), nausea (45%), vomiting (34%), asthenia (34%), fatigue (32%) and hypertension (26%). Discontinuation due to adverse events occurred in 17% of patients receiving sunitinib versus 8% placebo [23].

The use of systemic chemotherapy is recommended in advanced pNEN but not necessarily in small bowel NEN [24]. Patients with a Ki-67 of > 15%, have a higher probability of response to chemotherapy. Capecitabine with temozolomide is an option for lower grade NEN [24]. A randomized study comparing temozolomide monotherapy versus CAPTEM (capecitabine-temozolomide) in advanced G1-2 pNEN showed an ORR of 33.7 and 39.7%, PFS of 14.4 and 22.7 months and OS of 53.8 and 58.7 months, respectively [25]. Toxicity includes haematological (27.2%), gastrointestinal (8.3%) and cutaneous (3.2%) adverse events, with 16% grade 3-4 toxicity [26].

## Intralesional PV-10

Rose Bengal sodium (4,5,6,7-tetrachloro-2’,4’,5’,7’-tetraiodofluorescein) is a coal tar-derived small molecule that was originally used as red wool dye but later has been used in medicine as a topical diagnostic to detect conjunctivitis / keratitis [27]. PV-10 (10% Rose Bengal sodium injection) was subsequently developed as an anti-cancer agent as it was found to be cytotoxic and preferentially retained in tumour cells while rapidly cleared from surrounding normal tissue. It also had the ability to mount a tumour-specific immune response. Intralesional PV-10 has been shown to be an effective agent in animal models of hepatocellular carcinoma (Hepa1-6 murine tumour cell homografts) [28, 29], as it accumulates in lysosomes of cancer cells, causing lysosomal disruption leading to immunogenic cell death or apoptosis of PV-10 injected lesions while sparing normal tissue [30, 31]. Moreover, PV-10 may induce host-mediated immune responses in bystander lesions, as preliminary murine homograft studies have demonstrated both a response of PV-10 in injected liver lesions of primary HCC, colon or pancreas origin, and a bystander response in uninjected tumours [32, 33]. This bystander response is also observed in human studies, e.g., IL PV-10 for (sub)cutaneous lesions in metastatic melanoma and IL PV-10 for liver metastases in patients with metastatic uveal melanoma [34, 35].

**Fig. 1:**
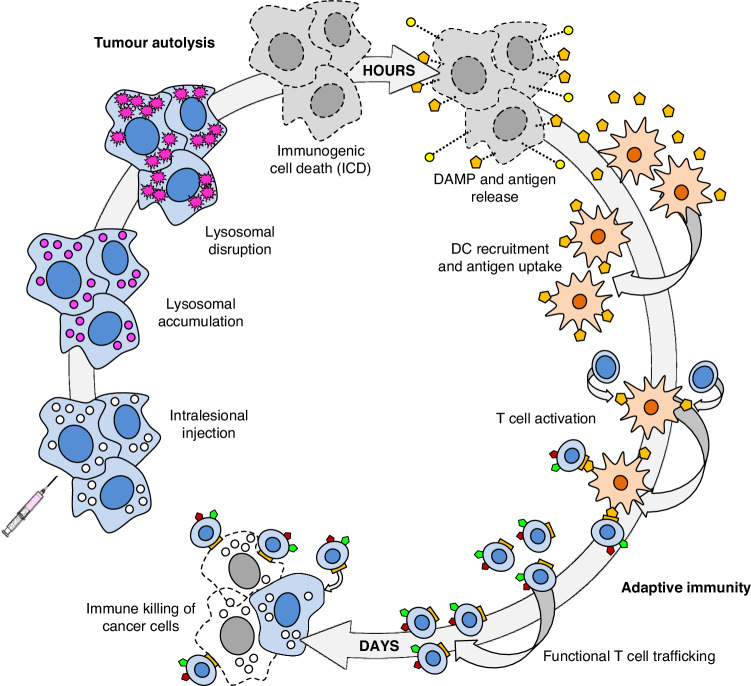
PV-10 Immuno-oncology cycle.

Non-clinical and clinical mechanism of action studies have shown that PV-10 may lead to significant increases in certain T-cell populations in peripheral blood within one to two weeks following tumour ablation. Liu et al. described an increased recruitment of dendritic cells in the tumour-draining lymph nodes via the damage-associated molecular pattern molecule (DAMP) HMBG-1, which can consequentially stimulate T cell proliferation [36]. (Fig. 1) An increase in functional CD8+ cells may be implicated in spontaneous regression of uninjected lesions (i.e., ‘bystander response’) by causing tumour-specific T cell-mediated immunogenic cell death and disease-specific adaptive immunity [36, 37]. This immunomodulation raises the potential for not only a local impact of IL PV-10 therapy, but also a systemic response driven by immune cell population changes.

IL PV-10 can be administered under visual, tactile or ultrasound guidance to (sub)cutaneous malignancies, and under CT or ultrasound guidance to tumours of the liver. Phase 1 testing of (sub)cutaneous intralesional PV-10 in metastatic melanoma resulted in an ORR of 48% of target lesions and locoregional DCR in 76% of patients [38]. Moreover, 27% of patients experiencing response of target lesions also exhibited an objective response in noninjected bystander lesions [38, 39]. These results were confirmed in a subsequent single-arm phase 2 study [35].

Intralesional administration of PV-10 into hepatic tumours (including primary HCC and other solid cancers metastatic to the liver) showed PV-10 was generally well tolerated, although photosensitivity, transient elevation of transaminases, nausea, vomiting and chest pain have been reported [40].

In NEN patients with progressive disease after available standard treatment, additional treatment options are needed. Given the preliminary evidence of safety and potential efficacy of percutaneous IL PV-10 administration for treatment of liver metastases of HCC, colorectal adenocarcinomas, and other primary tumours, we explored intralesional PV-10 as a novel palliative therapy for advanced NEN.

## Methods

### Study design

This was a single centre, open-label study to evaluate the safety, tolerability, and efficacy of intralesional injection of PV-10 on tumour growth and symptomology in patients with neuroendocrine tumours metastatic to the liver.

Due to the limited safety data in NEN, the initial design of the study divided the patients into two cohorts. Cohort 1 (*n* = 6 pts) received IL PV-10 to a single lesion per treatment cycle (to a maximum dose of 15 mL of PV-10) to allow the study investigators to assess safety and tolerability before proceeding with Cohort 2. Patients in Cohort 2 (*n* = 6) could receive injection to multiple lesions per treatment cycle, provided that any preceding treatments with PV-10 were well tolerated and a period of six or more weeks after prior IL PV-10 injection was respected.

### Study population

Key eligibility criteria included patients aged 18 years or older with progressive liver metastases of functional NEN that were not amenable to resection or other potentially curative therapies. Patients were required to have an ECOG (Eastern Cooperative Oncology Group) performance score of 2 or less and experience at least one of the following NEN-related symptoms: flushing, diaphoresis, diarrhoea, abdominal discomfort, hyperacidity, dyspnoea or palpitations. Eligible target lesion(s) were 1.0 to 3.9 cm in longest diameter (as determined by previous safety studies) [41] and amenable to percutaneous injection as determined by the study’s interventional radiologist. All targets were required to overexpress somatostatin receptor (SSTR) on baseline [^68^Ga]Ga-DOTA-octreotate PET-CT. Patients on long-acting somatostatin analogues were required to be clinically stable and somatostatin analogues were to be continued throughout the study period.

Other concurrent oncological treatments such as ablation modalities (e.g., cryotherapy, radiofrequency ablation), surgery, chemotherapy or radiation, were not permitted four weeks prior or during the IL PV-10 administration cycle.

Patients with significant concurrent illness (eg. unstable cardiovascular, cerebrovascular, renal, gastrointestinal, pulmonary or endocrine disorders) were not allowed to participate. Also, patients who had received agents posing a clinically significant risk of photosensitivity reaction within five half-lives of IL PV-10 administration were excluded.

### Study procedures

The study drug PV-10 was supplied as a sterile, non-pyrogenic saline solution containing 10% rose bengal sodium with 0.9% saline in 5 mL single use vials. Each patient received a dose of PV-10 into a single target lesion (Cohort 1) or multiple target lesions (Cohort 2) under ultrasound guidance, with the amount of IL PV-10 administered proportional to the size of the target lesion as determined via an algorithm.

Immediately following the procedure, a plain CT of the liver was performed to localise the radio-opaque PV-10 in the injected lesion. Patients were admitted for routine observation for 23 hours following IL PV-10 injection. Adverse events were graded by the Common Terminology Criteria for Adverse Events v4.0 (CTCAE). If patients had treatment-related abnormalities of CTCAE grade 3 or higher, or gross evidence of PV-10 in skin or urine (i.e., red or pink discoloration) at the end of the 23-hour observation period, they were kept under observation in the hospital with adequate support until the abnormalities normalized to less than grade 2 and any gross discoloration of skin or urine abated. Patients were advised to avoid exposure to sunlight for 48 h and were instructed to cover their arms, legs, and head on discharge from the hospital to minimize the risk of photosensitivity reactions. Furthermore, the skin had to be protected from prolonged exposure to direct sunlight (i.e., more than a few minutes) for two weeks after IL PV-10 administration. Concomitant medications posing an increased risk of photosensitivity (e.g., thiazide diuretics) were to be avoided for one week following IL PV-10 administration.

Laboratory tests (i.e., complete blood count, comprehensive metabolic panel including liver function tests (LFTs), coagulation and thyroid function) and vital signs (i.e. temperature, heart rate, blood pressure, respiratory rate) were performed at screening and at days 1, 7 and 28 following PV-10 injection. LFTs were also performed on Day 3. These assessments were repeated at long-term follow-up of three months and six months after IL PV-10 injection.

Patients were eligible to repeat the study procedure if they were deemed to have tolerated the first procedure and another target lesion was identified.

Objective response of target lesions and bystander lesions (if present) were evaluated according to two-dimensional European Association for the Study of the Liver (2D EASL) criteria from contrast-enhanced helical CT performed at six weeks and [^68^Ga]Ga-DOTA-octreotate PET-CT at three months after PV-10 injection. Lesional ^68^Gallium DOTATATE PET standardised uptake value (SUV) were measured to assess the effect of PV-10 on somatostatin receptor (SSTR) density of target lesions. The maximum SUV (SUV_max_) was calculated by automatically drawing around the target lesion. SUV_max_ of the lesions relative to the maximal splenic uptake was calculated by dividing the SUV_max_ of lesions by the SUV_max_ of the spleen (SUV_T/S_) [42]. Any decrease in the SUV_max_ and SUV_T/S_ in the PV-10 injected lesions was considered a positive response to the treatment. We also measured the peak SUV of target lesions corrected for lean body mass (SUL_peak_) to assess the SSTR response, adapted from PET Response Criteria in Solid Tumours (PERCIST) version 1.0 [43].

### Study outcome measures

The primary endpoint of the study was safety. Any expected or unexpected complications during the study from time of consent to six months were recorded on case-report forms. Serious adverse events were reported to the local HREC as per institutional requirements. Secondary endpoints included ORR (per RECIST 1.1) assessed by contrast enhanced CT (2D EASL criteria) and standardised uptake value (SUV) response on ^68^Ga-DOTA-octreotate PET-CT at 3 months; biochemical response (Chromogranin A (CgA) or 5-HIAA, lymphocytes) and quality of life assessed by patient-reported outcomes using the EORTC QLQ-C30 and GI.NET21 QOL instruments at baseline, 1 month and 3 months.

### Statistical analysis

The study results were analysed by descriptive statistics, as rigorous inferential statistics were not feasible due to the small treatment group sample size and the non-randomized nature of the study. As there was no difference in study procedures between Cohort 1 and Cohort 2, these patient results were pooled. ORR was determined. Overall survival (OS) and progression-free survival (PFS) were estimated by use of the Kaplan-Meier method. Sub-group analyses were performed for tumour type, tumour grade and number of previous treatment lines.

The statistical analysis for [^68^Ga]Ga-DOTA-octreotate PET-CT response evaluation was performed by using https://real-statistics.com. P-value was assessed by using Wilcoxon signed-rank test for paired samples and box plot with outliers were done for distribution of data to assess the change in SUV measurements.

## Results

Fourteen patients underwent screening procedures. One patient was ineligible due to clinical decline with ECOG score > 2 on progressive disease and one patient had lesions larger than the size limit. Twelve patients with progressive liver metastatic neuroendocrine tumours were enrolled between 2016 and 2021.

There was a 1:1 sex ratio and a median age of 66 years (range 47–79 years). (Table 1) The primary tumour sites were 7 small bowel (58%), 4 pancreas (33%) and 1 caecal (8%). The pathological grades were grade 1 (25%) and 2 (66.6%); no patients with grade 3 disease were treated. All patients had radiographic progression of disease at time of enrolment (documented as ≥ 20% increase in size per RECIST 1.1 criteria) and had received prior treatment with somatostatin analogues. Ten patients had prior PRRT treatment with [177Lu]Lu-DOTA-octreotate; two patients were ineligible for PRRT due to FDG/DOTATE PET mismatch (*n* = 1) and high risk of toxicity in low tumour burden (*n* = 1). The median CgA value was 1585 μg/L (range 35–10370 μg/L). One lesion was injected per procedure for each of 12 patients as none of the patients in cohort 2 were deemed suitable for multiple injections per cycle by the interventional radiologist. The majority of patients (*n* = 8) received 1 injection cycle in total, with multiple treatments delivered in a small number of eligible patients: 2 cycles (*n* = 2), 3 cycles (*n* = 1) and 4 cycles (*n* = 1). The median dose of PV-10 delivered per cycle was 1.9 ml (range 0.5–8.7 ml).

**Table 1:** Baseline patient characteristics.

Study ID	Sex	Age	Location of primary tumor	Grade	Time since diagnosis (months)	Prior treatment lines	Prior PRRT	Number of PV-10 cycles	Response CT	Treatment free interval (months)	OS after PV-10 injection (months)
0101	Male	63	Small bowel	G2	158	4	Yes	4	SD	N/A	41
0102	Female	65	Small bowel	G1	146	5	Yes	2	SD	3	22
0103	Female	47	Pancreas	G1	16	2	Yes	1	SD	11	Alive at 77 months
0104	Male	72	Caecum	G1	150	1	No	1	SD	N/A	Alive at 75 months
0105	Male	58	Small bowel	G2	65	2	Yes	1	PR	N/A	5
0106	Male	72	Pancreas	G2	91	7	Yes	1	PD	-	5
0107	Female	79	Small bowel	-	161	4	Yes	1	SD	3	9
0108	Female	75	Small bowel	G2	77	4	Yes	1	SD	-	25
0109	Female	68	Pancreas	G2	22	2	No	1	SD	6	16
0112	Male	75	Small bowel	G2	71	3	Yes	3	SD	3	37
0113	Male	65	Pancreas	G2	59	2	Yes	1	PD	N/A	7
0114	Female	61	Small bowel	G2	28	2	no	2	SD	16	Alive at 50 months

*PD* progressive disease, *PR* Partial response, *SD* Stable disease,

*N/A* no other treatments have been administered after IL PV-10

- : no data available.

Patient 0107 histopathology back dates from 2005, before current WHO classification was used. Unfortunately, no data is available on the tumor grade.

As treatment-emergent adverse events, we report grade 1/2 transient post-procedure pain at the injection site in 11 patients (91.6%). (Table 2) A grade 3 photosensitivity reaction developed in one patient and face oedema in one patient. Elevated hepatic enzymes were described with grade 1-2 elevation in three patients and grade 3 in one patient. A carcinoid flare occurred in 2 patients (16%) and increased flushing in 6 patients (50%). Grade 1-2 chromaturia was noted in 5 patients (41.6%) and discoloured faeces in 6 patients (50%). Grade 3 hypertension was observed in two patients (16%).

**Table 2:** Treatment-Emergent Adverse Events.

System Organ Class Preferred Term^1^	Treatment-Emergent Adverse Events: *N* (%)^2,3^ (*N* = 12 subjects)^4^			
	CTCAE Grade^5^			
	1	2	3	All
General disorders and administration site conditions				
Injection site pain	3 (25%)	8 (66.7%)	0 (0%)	11 (91.7%)
Flushing	3 (25%)	3 (25%)	0 (0%)	6 (50%)
Fatigue	4 (33.3%)	0 (0%)	0 (0%)	4 (33.3%)
Face oedema	0 (0%)	0 (0%)	1 (8.3%)	1 (8.3%)
Gastrointestinal disorders				
Faeces discoloured	6 (50%)	0 (0%)	0 (0%)	6 (50%)
Constipation	2 (16.7%)	0 (0%)	0 (0%)	2 (16.7%)
Diarrhoea	2 (16.7%)	0 (0%)	0 (0%)	2 (16.7%)
Nausea	2 (16.7%)	0 (0%)	0 (0%)	2 (16.7%)
Investigations				
Alanine aminotransferase increased	1 (8.3%)	1 (8.3%)	0 (0%)	2 (16.7%)
Aspartate aminotransferase increased	1 (8.3%)	0 (0%)	1 (8.3%)	2 (16.7%)
Renal and urinary disorders				
Chromaturia	5 (41.7%)	0 (0%)	0 (0%)	5 (41.7%)
Skin and subcutaneous tissue disorders				
Rash	2 (16.7%)	0 (0%)	0 (0%)	2 (16.7%)
Photosensitivity reaction	0 (0%)	0 (0%)	1 (8.3%)	1 (8.3%)
Vascular disorders				
Hypertension	0 (0%)	0 (0%)	2 (16.7%)	2 (16.7%)
Neoplasms benign, malignant and unspecified (incl cysts and polyps)				
Carcinoid syndrome	1 (8.3%)	1 (8.3%)	0 (0%)	2 (16.7%)
Hepatobiliary disorders				
Biliary obstruction	0 (0%)	0 (0%)	1 (8.3%)	1 (8.3%)

^1^System Organ Class (SOC) and Preferred Term (PT) of each adverse event coded per the MedDRA^®^ v. 27.0 terminology dictionary.

^2^Treatment-emergent adverse events (TEAEs) are all adverse events with an onset on or within 28 days following the date of any dose of PV-10, or that occur any time during the study interval and are deemed by the investigator to be at least possibly related to the study drug or treatment, including any additional study treatment administered during the study interval.

^3^Events occurring in more than one study participant, or with Grade 3 or higher severity, are presented.

^4^Data from all participants in study PV-10-NET-01 (*N* = 12).

^5^Adverse event grade per CTCAE v. 5.0. If a subject experienced an AE more than once during the study the greatest severity is presented.

**Fig. 2:**
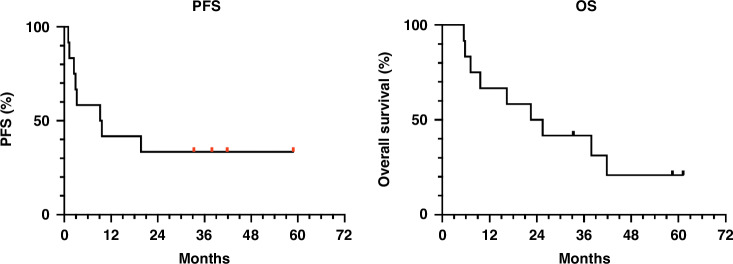
Kaplan-Meier curves of PFS and OS in general population.

**Fig. 3:**
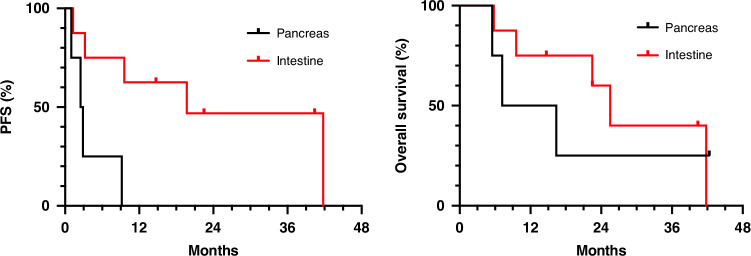
Kaplan-Meier curves of PFS and OS in pancreas NEN and small bowel NEN subtypes. PFS progression free survival, pNEN pancreatic neuroendocrine neoplasm, OS overall survival, SI-NEN small intestine neuroendocrine neoplasm. The median PFS (left) was 2.7 months in pNEN versus 19.7 months in SI-NEN. The median OS (right) was 11.8 months in pNEN versus 25.5 months in SI-NEN.

There was an ORR in 42% of the injected lesions on CT 6 weeks post injection. An overall DCR was achieved in 84% of patients (8.3% partial response and 75% stable disease), with matching stable CgA in 10 patients (83%). The median PFS (mPFS) was 9.4 months (range 1.0–58.8 months) and median OS (mOS) 24.0 months (range 5.5–61.2 months) (Fig. 2). When looking at separate tumour types, a mPFS of 2.7 months versus 19.7 months and mOS of 11.8 months versus 25.5 months is described in pancreatic NEN and small bowel NEN (SI-NEN) respectively (Fig. 3). In patients that were heavily pretreated, mPFS was 9.2 versus 9.6 months and mOS of 41.8 versus 22.5 months in patients ≤ 2 previous treatment lines and patients > 2 lines respectively. We observed a better mPFS and mOS in patients with grade 1 versus grade 2 disease.

By using PERCIST version 1.0 criteria, a partial metabolic response (PMR) was seen in 33.3% and stable metabolic disease (SMD) in 55.5% of injected lesions, with overall DCR seen in 88.8% of lesions. We observed a reduction in median SUV_max_ (from 20.5 to 16.02), SUL_peak_ (from 17.4 to 13.1) and SUV_T/S_ (from 1.5 to 1.1) from baseline to post IL PV-10 injection administration. *P*-value was statistically significant for SUL_peak_ (*p* = 0.030), but not significant for SUV_max_ measurements (*p* = 0.1) in post-therapy studies.

The health-related quality of life (HRQOL) assessment showed stable or improved endocrine symptoms and global health status in 8 of 11 patients (73%) at one month, and in 6 of 10 patients (60%) at three months.

An upregulation of NK cells (CD3-CD56 + ) and activated CD4 + T cells (CD3 + CD56-CD4 + CD25 + ) was observed in peripheral blood collected 7–28 days post IL PV-10 administration.

## Discussion

IL PV-10 was administered safely in 12 patients with mNEN who have had multiple prior treatment lines, including somatostatin analogues (*n* = 12) and PRRT (*n* = 10).

The observed toxicity was consistent with previous IL PV-10 clinical trials with hepatic lesions of different primaries and consisted mainly of pain at injection site, photosensitivity rash and transient elevated liver enzymes. The liver enzymes resolved spontaneously by day 7 [44]. We also observed grade 3 hypertension in two patients, possibly provoked by pain. As Rose Bengal is a dye, it can cause temporary pink discoloration of the urine and faeces (observed in about 50% of patients) when systemic uptake and subsequent excretion via urine and bile is present. Cutaneous accumulation of Rose Bengal can lead to photosensitivity reactions when activated by exposure to visible and ultraviolet light. These reactions can range from mild to severe, with possible facial oedema. Prophylactic measurements to prevent light exposure was recommended and severe reactions were treated with systemic corticosteroids and antihistamines. Transient grade 1–2 flushing was observed in 6 patients (50%). This might be explained by release of mediators in the blood stream causing a carcinoid syndrome flare. All patients who experienced a flare had previously reported flushing as part of their functional syndrome symptoms. Most flares were brief (lasting a few minutes) and all flares improved within 48 h after Octreotide 100 µg SC injection. All observed adverse events resolved; no patients discontinued the study due to a treatment-emergent adverse event.

An ORR of 42% for PV-10 injected lesions with overall DCR in 83% in this heavily pretreated group of patients was seen. Together with mPFS of 9.4 months and mOS of 24.0 months, there is evidence for activity of IL PV-10 in grade 1–2 mNEN. When looking at the OS curve, we observe a long tail with 3 out of 12 patients still alive at time of data analysis.

We demonstrate a trend of longer PFS and OS in small bowel NEN compared to pancreatic NEN, with a mPFS of 19.7 versus 2.7 months and mOS of 25.5 versus 11.8 months, respectively. However, due to the small patient numbers (7 SI-NEN, 4 pNEN), more data are necessary to confirm this trend. A possible explanation could be found in the difference in natural evolution and response to IL PV-10 treatment of mNEN of varying primary sites, with neuroendocrine tumour of pancreatic origin tending to be a poor prognostic feature. In a retrospective study using the SEER database, it was shown that overall pancreatic NEN had the lowest mOS compared to other primary tumour sites [45], with grade 1-2 small intestine NEN having the best mOS of 103 months compared to a mOS of 60 months in grade 1-2 pancreas NEN. Another theory is that a greater immune response is yielded in small bowel NEN compared to pancreas NEN, however, a trial by the Dana-Farber Cancer Institute investigating archival NEN tumour specimens seems to contradict this theory as they observed larger presence of T-cell infiltrates in the extratumoural compartment pNEN than in small bowel NEN and a similar low PDL1 expression in both cancer subtypes [46].

### Comparison to IL PV-10 response in different tumour types

The response rates are similar to those described in prior phase I trials with IL PV-10 treatment in metastatic melanoma, where a ORR of 48% and DCR 76% were reported [38]. A follow-up phase II trial wielded an ORR of 61% and DCR of 79%, with a mPFS of 8.5 months [35]. A different trial in 57 patients with in transit melanoma metastases showed an ORR of 87% after sequential IL PV-10 injections, with a mOS of 25 months [47].

### Comparison of efficacy IL PV-10 to currently available systemic therapies

With an ORR of 42% for PV-10 injected lesions, IL PV-10 has a higher response rate than everolimus (ORR 18%) [19], sunitinib (ORR 9.3%) [23] or CAPTEM (ORR 39.7%) [26]. Also, DCR seems improved when comparing to everolimus (73%), sunitinib (72%) and CAPTEM (77%). Despite the higher ORR and DCR, IL PV-10 has a shorter mPFS. This might be explained by a wear-off effect, as liver lesions could only receive a maximum of one single IL PV-10 injection per lesion, whereas other systemic therapies are ongoing until disease progression.

### Health-related quality of life

A systematic review from 2022 investigated 61 studies on HRQOL [48]. So far, all systemic therapies showed no worsening in HRQOL, however only the NETTER-1 study has been able to show a statistically significant improvement in HRQOL in patients with small bowel NEN treated with [^177^Lu]Lu-DOTA-octreotate versus high-dose octreotide [49].

In this study, all included patients had functional NEN with at least 2 NEN-related symptoms. About 60% of patients reported a stable or improved global health status after 3 months. Importantly, endocrine symptoms seemed to be managed better with IL PV-10 treatment, with 50% of patients reporting improvement and 16% of patients reporting stable symptoms after 3 months. Approximately 50% of patients reported a decrease in GI symptoms. Overall, the improved global health status and symptom control supports systemic effect of IL PV-10 and clinical benefit.

### Costs

Although a health cost analysis is not possible in this small phase 1 trial, PV-10 is an easily accessible and a relatively inexpensive product compared to current standard treatment modalities. Any future analysis would need to consider hospital admission and procedural costs when comparing to systemic therapy, however, when comparing to PRRT with ^177^Lu-DOTATATE the associated hospital admission costs beyond the used agent may be similar.

### Future clinical trials

NEN have previously been described as “immunologically cold tumours” that respond poorly to anti-PD(L)1 monotherapy. Approximately 30% of patients have PD-L1 positive NEN [50, 51]. According to KEYNOTE – 028, pembrolizumab monotherapy showed limited antitumour activity in pretreated PDL-1 positive NEN with an ORR of only 12% in carcinoid tumours and 6.3% in pancreatic NEN [52]. Stable disease lasting more than 6 months occurred in 32% and 31% of patients, respectively.

As PV-10 stimulates the immune response, it would be interesting to further investigate the combination of IL PV-10 and systemic anti-PD(L)1 therapy in NEN tumours. It is known that PV-10 can elicit a local and systemic immune response after injection and can result in regression of both injected target lesions as well as un-injected non-target lesions [27]. Previous studies have shown that PV-10 increases the CD8 + , CD4 + , CD3 + T cells and NK cells in peripheral blood and it was thought that through this mechanism PV-10 can cause a ‘bystander response’[36, 37]. In this trial, an upregulation in activated CD4 + T cells and NK cells in peripheral blood was also noted, consistent with results in other solid tumour types, however, only one out of 12 patients had a bystander response on restaging. This supports the theory of a potential benefit of combination therapy of PV-10 and immune checkpoint inhibitors in mNEN patients to investigate whether PV-10 may be a new approach to change this immunologically “cold tumour” into a “hot tumour”[53, 54].

In a phase 1b/2 trial in which 23 patients with immune checkpoint-naïve advanced cutaneous melanoma received PV-10 in combination with pembrolizumab, an increase in ORR up to 67% and estimated mPFS of 11.7 months was achieved [55]. Median OS was not reached. There are plans for a Phase 2 study combining PV-10 and Keytruda (pembrolizumab) for treatment of metastatic neuroendocrine tumours.

Another potential combination therapy that can be explored is the combination of PV-10 with PRRT. Preclinical trials with murine models have shown that PRRT increases tumour infiltration by CD49b + /FasL+ NK cells [56]. A trial of Esfahani et al. investigated the combination of PRRT and pembrolizumab in mice implanted with gastroenteropancreatic NEN and concluded that combination of PRRT and anti-PD1 shows a higher inflammatory response (specifically higher CD8+ effector T cells) to NEN and a better overall outcome than immune checkpoint inhibition or PRRT alone [57].

Also, we could consider sequential IL PV-10 to previously injected lesions in patients who have stable disease or partial response on restaging imaging to maximize IL PV-10 effects and maintain disease control over a longer period of time.

### Limitations

This trial does have limitations. As this phase I trial was designed to assess safety / toxicity, only a small number of patients (*n* = 12) were included, hence it is difficult to draw general conclusions on the secondary endpoint ORR. This study included mainly patients with small bowel NEN (58%) and hence we have limited data on pancreatic or lung NEN. Also, only a small patient population (*n* = 6) underwent long term follow-up in this phase I trial, emphasizing the necessity for further research with larger sample sizes.

We have noticed a wide range in delivered IL PV-10 dose per cycle, from 0.5 to 8.7 mL. In the study design, patients were allowed to be administered a dose of 0.50 mL PV-10 per cm^3^ lesion volume with a maximum PV-10 dose of 15 mL (equal to 1500 mg of Rose Bengal), based on previous safety studies. Although this dose is calculated based on target lesion size, future investigations could investigate whether higher total doses might possibly yield a larger systemic immune response.

Also, concurrent somatostatin analogues might be a potential source of interference with [^68^Ga]Ga-DOTA-octreotate uptake. A variable relationship exists between timing of long acting somatostatin analogues and [^68^Ga]Ga-DOTA-octreotate PET-CT. A small cohort, prospective trial has investigated this potential bias and noticed diminished (background) uptake of [^68^Ga]Ga-DOTA-octreotate in liver, spleen and thyroid but no significant differences in uptake for primary tumor or metastatic lesions [58]. Dedifferentiation of a lesion may result in the loss of SSTR expression, therefore a decrease in SSTR uptake does not necessarily reflect a decrease in lesion viability. FDG PET was not performed to assess metabolic response, as NENs are typically poorly FDG avid.

## Conclusion

Intralesional PV-10 chemotherapy elicited no safety concerns and multiple cycles were delivered safely in suitable patients.

Encouraging evidence on disease control and improvement in HRQOL was noted in a heavily pre-treated, grade 1-2 mNEN population. Intralesional PV-10 has the potential for both local control of injected lesions and ‘bystander response’ of uninjected lesions, most likely related to upregulation of the adaptive immune system. The adaptive immune upregulation observed in mNEN is consistent with other solid tumours and supports the potential systemic benefit of combination with immune checkpoint inhibitors to potentiate a stronger anti-tumour immune activity.

## Data Availability

The datasets generated and analysed during the current study are available from the corresponding author on reasonable request.
